# Developmental and evolutionary novelty in the serrated teeth of theropod dinosaurs

**DOI:** 10.1038/srep12338

**Published:** 2015-07-28

**Authors:** K. S. Brink, R. R. Reisz, A. R. H. LeBlanc, R. S. Chang, Y. C. Lee, C. C. Chiang, T. Huang, D. C. Evans

**Affiliations:** 1 Department of Biology, University of Toronto Mississauga, 3359 Mississauga Rd., Mississauga, Ontario, L5L 1C6, Canada; 2 Department of Ecology and Evolutionary Biology, University of Toronto, 25 Willcocks St. Toronto, Ontario, M5S 3B2, Canada; 3 Department of Optics and Photonics, National Central University, Jhongli City, Taoyuan 32001, Taiwan; 4 National Synchrotron Radiation Research Center, Hsinchu Science Park, Hsinchu 30076, Taiwan; 5 National Chung Hsing University, Taichung City 402, Taiwan; 6 Royal Ontario Museum, 100 Queens Park, Toronto, Ontario, M5S 2C6, Canada

## Abstract

Tooth morphology and development can provide valuable insights into the feeding behaviour and evolution of extinct organisms. The teeth of Theropoda, the only clade of predominantly predatory dinosaurs, are characterized by ziphodonty, the presence of serrations (denticles) on their cutting edges. Known today only in varanid lizards, ziphodonty is much more pervasive in the fossil record. Here we present the first model for the development of ziphodont teeth in theropods through histological, SEM, and SR-FTIR analyses, revealing that structures previously hypothesized to prevent tooth breakage instead first evolved to shape and maintain the characteristic denticles through the life of the tooth. We show that this novel complex of dental morphology and tissues characterizes Theropoda, with the exception of species with modified feeding behaviours, suggesting that these characters are important for facilitating the hypercarnivorous diet of most theropods. This adaptation may have played an important role in the initial radiation and subsequent success of theropods as terrestrial apex predators.

Carnivorous tetrapods exhibit varying degrees of dental specializations for grasping and tearing their prey items, including ziphodonty– blade-shaped teeth with well-developed serrated cutting edges^1^. The presence of serrations (or denticles) on teeth usually indicates a carnivorous diet, based on extant Varanidae^2^, and practical assays and morphometric analyses of teeth^3^,^4^,^5^,^6^. Ziphodont teeth evolved sporadically and convergently in extinct vertebrate groups, including early synapsids^7^,^8^, marine and terrestrial crocodilians^9^,^10^, and early archosaurs, including phytosaurs^11^ and dinosaurs^3^. However, among toothed theropod dinosaurs, ziphodonty is nearly ubiquitous. Previous histological study of ziphodonty in the teeth of the tyrannosaurid *Albertosaurus* revealed unusual enamel cracks leading to a void in the dentine called an “ampulla” between adjacent denticles along the distal carinae, which were hypothesized to have dissipated stresses on the tooth during feeding^4^,^5^,^6^. Given that feeding specializations have been suggested as important in the radiation of dinosaurs^12^,^13^, the evolution of these unusual dentinal structures, if they are plesiomorphic for Theropoda, might have been an important functional innovation linked to their initial evolutionary radiation and subsequent success as apex predators during the Mesozoic. Our systematic survey of the tissue-level organization of ziphodont teeth across a broad phylogenetic scope of theropod species and closely related outgroups using histological thin-sections, Scanning Electron Microscopy (SEM), and Synchrotron Radiation Fourier Transform Infrared spectroscopy (SR-FTIR) allow us to reject the hypothesis that the “ampulla” originated through stress on the tooth, and propose a new developmental model based on reptilian tooth development^14^,^15^,^16^ to explain the unique microanatomy of ziphodont dinosaur teeth.

## Results

### Erupted teeth

**Figure 1:**
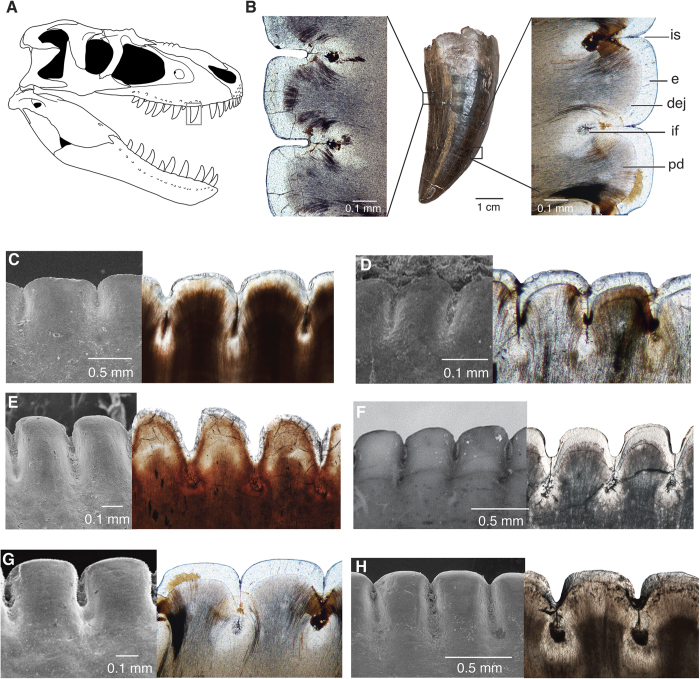
Microanatomy of ziphodont archosaur teeth with deep interdental folds. (**A**) Skull of *Gorgosaurus libratus*, a tyrannosaurid dinosaur, drawn by Danielle Dufault. (**B**) Complete tooth (ROM 31535) and sagittal thin sections through distal and mesial carinae of a maxillary cf. *Gorgosaurus* sp. (ROM 57981) tooth. (**C–H**), carinae of ziphodont teeth under SEM (left) and in thin section (right). (**C**), Indeterminate phytosaur, ROM 7981, mesial carina. (**D**) *Coelophysis bauri*, CM 76863, distal carina. (**E**) *Allosaurus fragilis*, ROM 66106, distal carina. (**F**) *Carcharodontosaurus saharicus*, ROM 52017 (left) and ROM 47691 (right), mesial carina. (**G**) cf. *Gorgosaurus libratus*, ROM 57981, mesial carina. (**H**) *Tyrannosaurus rex*, ROM 66108, mesial carina. Abbreviations: dej, dentine-enamel junction; e, enamel; if, interdental fold; is, interdental sulcus; pd, primary dentine.

**Figure 2:**
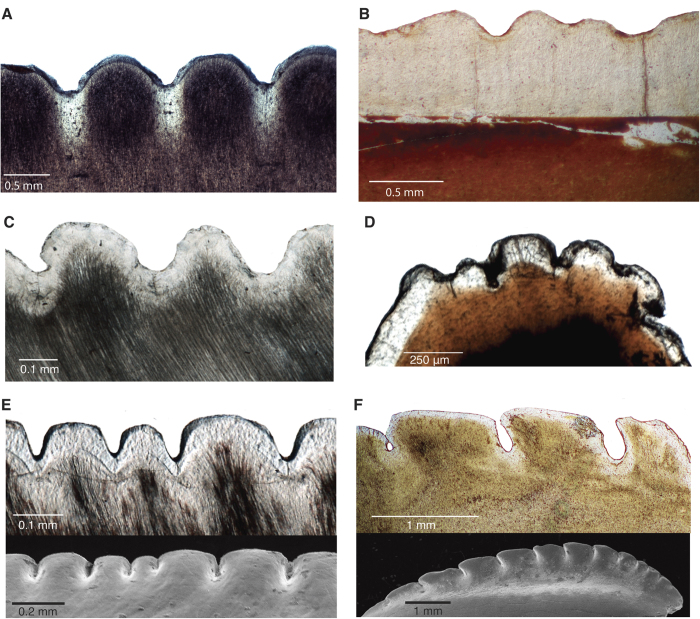
Microanatomy of ziphodont teeth lacking deep interdental folds. (**A**) *Carcharodon megalodon* (ROM 30530). (**B**) *Smilodon* sp. (ROM 3288) carina, lacking denticles (not a true ziphodont animal as the carina is composed of enamel only). (**C**) *Varanus komodoensis* (ROM R7565), distal carina, with unevenly sized denticles. (**D**) Hadrosaurid (ROM 58205) labial denticles. (**E**) Sagittal thin section through the distal carina of a maxillary tooth of *Dimetrodon grandis* (ROM 6039), showing inconsistently-sized denticles. Thin section (top), SEM (bottom). (**F**) *Troodon formosus* (ROM 05089), mesial carina. Thin section (top), SEM (bottom).

Erupted homodont maxillary teeth (Fig. 1A) show a level of tissue organization in the serrated cutting edge that is common to archosaurs. Deep folds are present in the dentine at the base of each externally visible interdental sulcus, reminiscent of the dentine folds present in teeth with tightly-folded plicidentine^17^, effectively increasing the size of the individual denticles inside the tooth on both the mesial and distal carinae (Fig. 1). The denticles in all theropod teeth have similarly sized denticle bases along the length of the carina that are tightly apposed (Fig. 1), but denticle sizes vary in ziphodont teeth in taxa that lack deep folds (Fig. 2).

**Figure 3:**
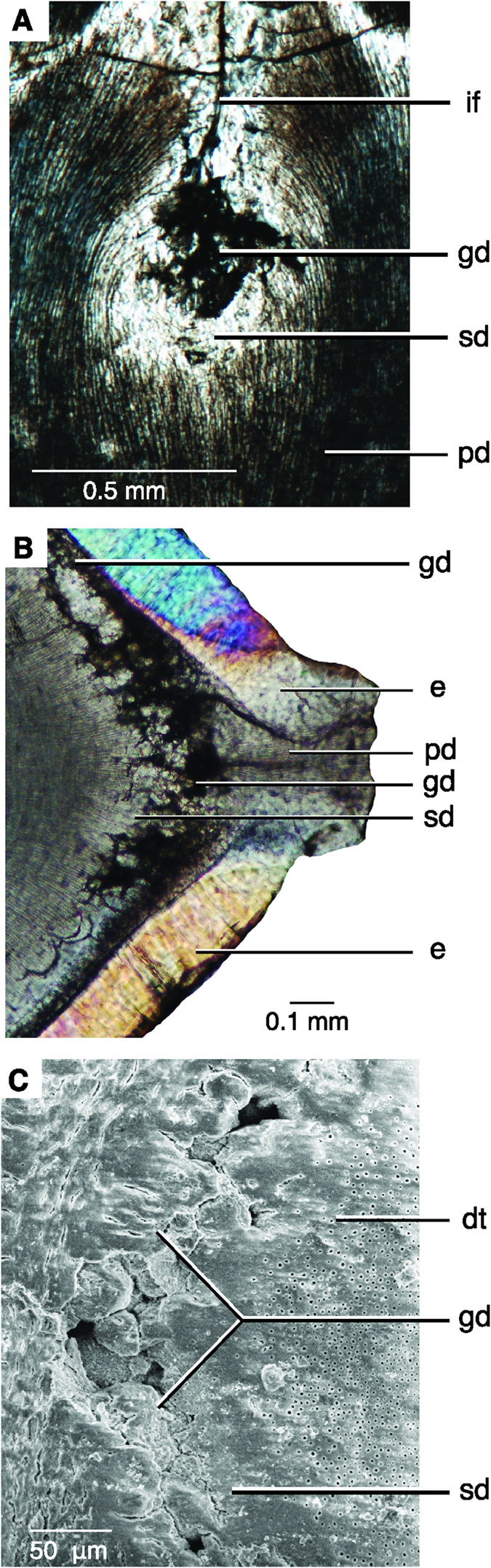
Microanatomy of interdental folds. (**A**) Sagittal thin section of an interdental fold below the DEJ in *Carcharodontosaurus saharicus* (ROM 52037). Carina to the top. (**B**) Oblique transverse thin section through an interdental fold and denticle in a maxillary tooth of *C. saharicus* (ROM 52037) under cross-polarized light. Carina to the right, pulp cavity to the left. (**C**) SEM image of an interdental fold in a cf. *Gorgosaurus* (ROM 57981) maxillary tooth in coronal section. Abbreviations: dej, dentine-enamel junction; dt, dentine tubules; e, enamel; gd, globular dentine; if, interdental fold; pd, primary dentine; sd, sclerotic dentine.

**Figure 4:**
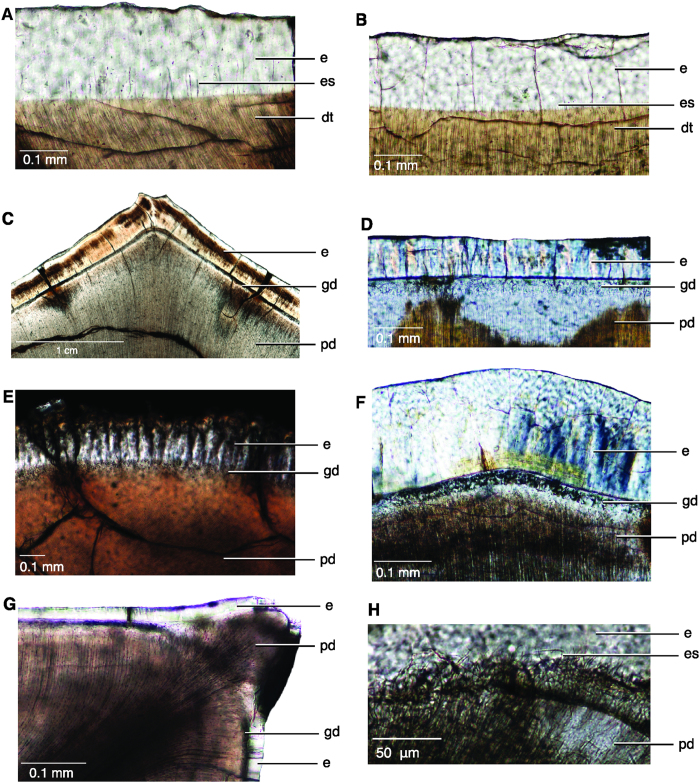
Dentine-Enamel Junction of archosaur teeth. (**A**) *Triceratops* sp. (ROM 67669) with enamel spindles and no globular mantle dentine. (**B**) Hadrosaurid (ROM 58205) with enamel spindles and no globular mantle dentine. (**C**) *Spinosaurus aegyptiacus* (ROM 65613) carina with dentine, globular dentine, and enamel ornamentations, but lacking true denticles. (**D**) Indeterminate phytosaur (ROM 7981) with globular mantle dentine. (**E**) cf. *Gorgosaurus* (CMN 2225) unerupted, newly forming tooth with globular mantle dentine, primary dentine, and incompletely mineralized enamel. (**F**) Cretaceous crocodilian (ROM 67512) with globular mantle dentine. (**G**) *Coelophysis bauri* (CM 87671) unerupted tooth, carina to the top right corner, showing a lack of globular dentine within the denticle. The globular dentine can be seen behind the dentine tubules of the denticle in the interdental space. (**H**) Sagittal thin section of the DEJ at a denticle tip in *C. saharicus* (ROM 52037), showing enamel spindles. Abbreviations: dt, dentine tubule; e, enamel, es, enamel spindle; gd, globular dentine; pd, primary dentine.

A number of features were found only in theropod dinosaurs. The mantle dentine immediately surrounding the interdental fold is globular in theropod teeth, and dentine tubules that originate in the fold and curve towards the pulp cavity extend outward from the globular dentine (Fig. 3A). In transverse and sagittal section, the globular dentine and interglobular spaces are visible as dark atubular areas surrounding the interdental fold (Fig. 3A,B). The distribution of globular dentine is unusual, being absent in each denticle, where enamel tufts and spindles are often present near denticle tips (Figs 3B and 4). The enamel tufts and spindles are similar to those observed in ornithischian dinosaurs (Fig. 4A,B). The appearance of globular dentine and interglobular spaces (0–50 μm), visible under SEM (Fig. 3C), are what was previously misinterpreted as the hollow “ampulla”^4^,^5^,^6^.

Surrounding the interdental fold is an area of clear dentine, originally thought to be a deep layer of enamel encircling the globular dentine^4^. We interpret this layer as sclerotic dentine, as the dentine tubules are transparent and not visible under SEM (Fig. 3).

The interdental fold is continuous below each interdental sulcus, forming a 1 μm channel through the enamel (Figs 1 and 3). The channel is identical at each interdental sulcus in an individual tooth, being similar in width, length, and orientation. The channel and interdental folds are present along the majority of the carina, but tend to be absent near the tooth apex where the tooth curves and the enamel is thickened. The structure of the DEJ (Dentine Enamel Junction) usually stops enamel cracks from penetrating into the dentine, thereby preserving the tooth from catastrophic damage^18^,^19^. Therefore, the presence of a channel through the enamel and the DEJ is unusual and unique to theropod dinosaurs.

### Unerupted teeth

**Figure 5:**
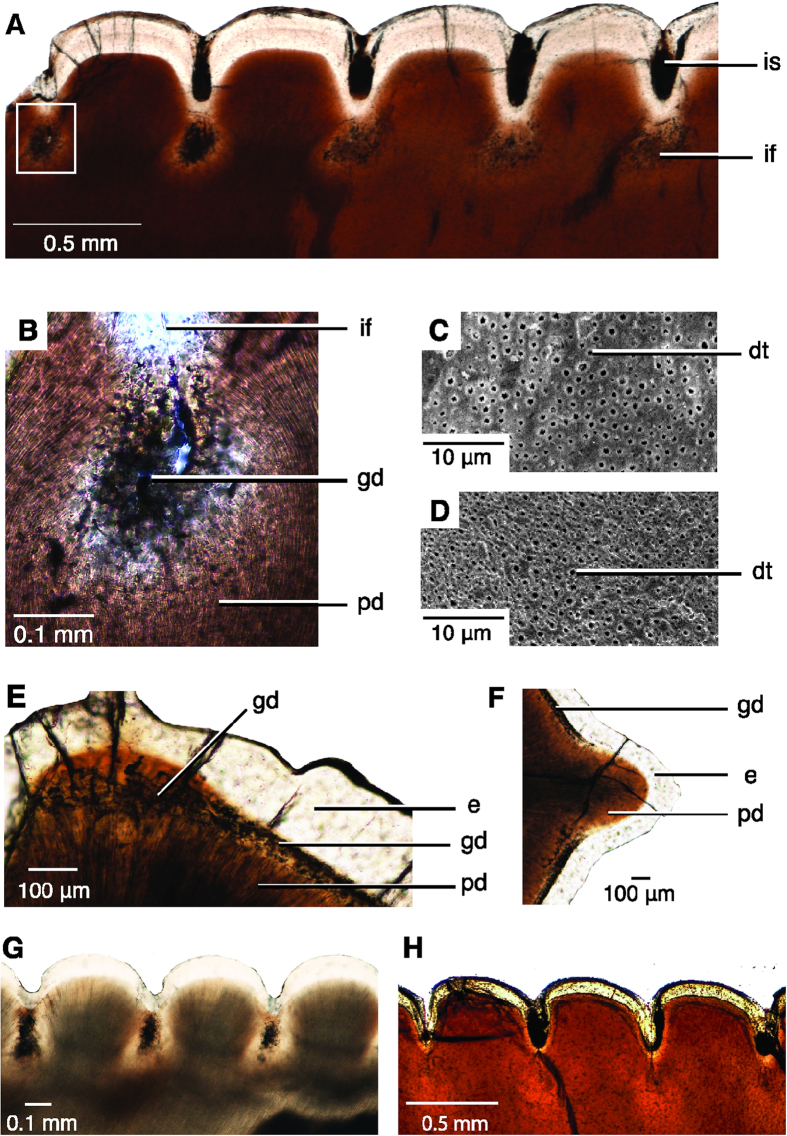
Microanatomy of unerupted cf. ***Gorgosaurus*** **sp. teeth.** (**A**) Distal carina of CMN 2225 showing the deep folds at the base of each interdental sulcus. (**B**) Detail of interdental fold from (A) showing absence of sclerotic dentine. (**C**) Dentine tubules in an erupted tooth (ROM 57981) showing peritubular dentine in each hollow tubule. (**D**) Dentine tubules in an unerupted tooth (CMN 2225) showing less peritubular dentine when compared to erupted tooth in (C). (**E**) Transverse section between two neighbouring denticles (CMN 2225), carina to the top. (**F**) Transverse section through a denticle showing absence of globular dentine in the denticle tip. Carina to the right. (**G**) Unerupted tooth of cf. *Gorgosaurus libratus* (RTMP 2012.012.0029), with interdental folds. (**H**) Unerupted tooth of *Allosaurus fragilis* (UMNH.VP 23761), with interdental folds. Abbreviations: dt, dentine tubule; e, enamel; gd, globular dentine; if, interdental fold; is, interdental sulcus; pd, primary dentine.

Histological sections of unerupted replacement teeth provide a developmental perspective on the microanatomy of theropod teeth. Unerupted teeth of *Allosaurus* and *Gorgosaurus* (Fig. 5) also have deep interdental folds. The folds are similar in shape to those of erupted teeth in being surrounded by globular mantle dentine, and extending to the external enamel surface through a channel in the interdental sulcus. However, the unerupted teeth lack the clear layer of sclerotic dentine that surround the interdental folds of erupted teeth (Fig. 5B). The dentine tubules are visible from the DEJ to the pulp cavity, even in the area of the fold. A comparison of the dentine tubules in the area of the fold shows deposition of thick peritubular dentine around each dentine tubule in erupted teeth only (Fig. 5C–D). Peritubular dentine deposition increases with age, and may be accelerated in response to an external stressor, forming sclerotic dentine^18^,^20^. The absence of sclerotic dentine is also noticeable in transverse section (Fig. 5E). As in erupted teeth, globular dentine is absent in the denticles, but is present directly below the DEJ in the remainder of the unerupted tooth (Fig. 5F).

### SR-FTIR spectroscopic analyses

**Figure 6:**
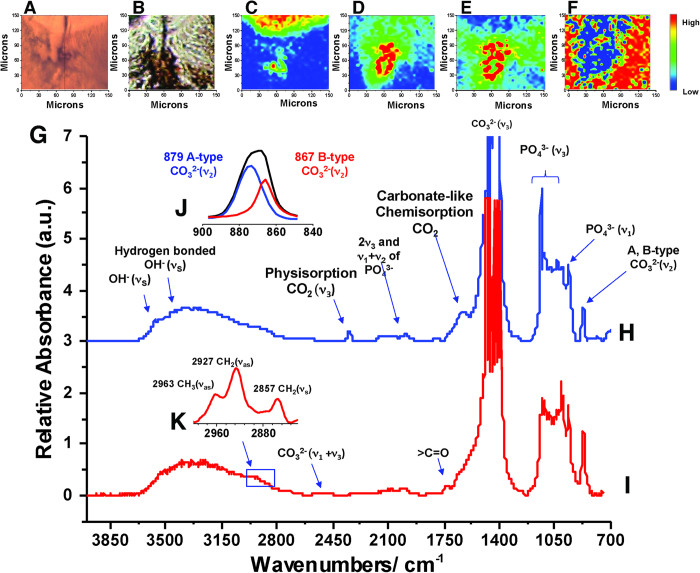
SR-FTIR spectra of enamel and dentine of a cf.*Gorgosaurus* tooth, ROM 30582. (**A–F**), region of interdental fold, (**A**) optical image, (**B**) reflectance image, (**C**) CO_2_ at 2345 cm^−1^ present in enamel and interdental fold. (**D**) cHAP-A at 879 cm^−1^ in the globular dentine of the interdental fold. (**E**) cHAP-B at 867 cm^−1^ in the globular dentine of the interdental fold. (**F**) Alkyl at 3000-2800 cm^−1^, representing organic material. Colour bar at the far right with blue as the lowest and red as the highest concentration. (**G**) FT-IR spectra and assignments. (**H**) FT-IR spectrum of enamel. (**I**) FT-IR spectrum of dentine. (**J**) Deconvolution of cHAP Type A and Type B of enamel. (**K**) Deconvolution of -CH_3_ and -CH_2_ of preserved organics in dentine.

SR-FTIR results support the interpretations made through SEM and histology (Fig. 6). The spectral assignment of type A and type B carbonate of carbonated hydroxylapatite (cHAP), being resolved after deconvolution of FTIR spectra, was in agreement with the results of previous investigations^21^,^22^,^23^. cHAP type A (Fig. 6D), considered to be invasive to the tooth, was found in the interglobular spaces around the interdental fold, and cHAP type B (Fig. 6E), considered to be native to the tooth, was found in the globular dentin. A noticeable organic signal within the primary dentine of the tooth was recovered, but no signal from the globular dentine (Fig. 6F,G). These findings were consistent with the results of previous investigations which indicated that the type A carbonate of cHAP was more energetically feasible and more thermodynamically stable than the type B carbonate of cHAP^24^,^25^. Thus, we propose that it was highly likely that carbonate from groundwater was substituted for the hydroxyl group of cHAP within the enamel to form type A carbonate, not affecting the dentine. The absence of an organic signal in the area of the interdental fold suggests that the interdental sulcus was likely not used to house bacteria for a septic bite in carnivorous dinosaurs^4^.

High levels of physisorbed CO_2_ (at 3574 cm^−1^ (combination band n3+2n2) and 2345 cm^−1^ (asymmetric stretching ν_3_)) were recorded within the globular dentine and the type A carbonate of the enamel, but not within the primary dentine (Fig. 6C). Also, the typical broad absorption band of chemisorbed CO_2_ was observed in the range of 1723-1586 cm^-1^, and its intensity was strongly related to the intensity of physisorbed CO_2_ within the tooth, consistent with previous studies^25^,^26^. We consider that CO_2_ was rarely produced from dissociation of carbonates of cHAP within the whole tooth because only the enamel layer presented the intense infrared absorption of physisorbed and chemisorbed CO_2_. Groundwater CO_2_ partial pressure is typically ~10–100 times higher than that of the atmosphere^25^, and higher levels of atmospheric CO_2_ have been suggested for the Mesozoic^26^. We therefore propose that the relatively higher level of CO_2_ was likely dissolved in the groundwater and infiltrated into the tooth for further reaction with the hydroxyl group of cHAP within the tooth tissues during fossilization.

The presence of CO_2_, cHAP type A, and cHAP type B, and a lack of organic signal in the area of the interdental fold confirm that the channel extending between the interdental fold and interdental sulcus was indeed open to the environment outside the tooth, and that the sclerotic dentine was successful in blocking the distal portion of the dentinal tubules adjacent to the globular dentine from the rest of the primary dentine of the tooth.

## Discussion

Models of tooth functionality and tooth wear^4^ have suggested that denticulate theropod teeth were efficient in “puncture and pull” feeding^27^, thereby introducing tension on the distal carina and compression on the mesial carina, necessitating a “kerf and drill” structure, or hollow “ampulla” only on the distal carina to prevent catastrophic breakage of the tooth^5^. However, interdental folds are present on both the mesial and distal carinae of erupted and unerupted teeth in all theropods examined, and the development of these structures begins before the tooth has erupted into the oral cavity. Moreover, the “ampulla” is not a hollow structure that might function as a “kerf and drill”, but is globular mantle dentine deposited at each interdental fold. We therefore reject the hypothesis that these structures are epigenetically formed with use of the tooth to counter tensional forces and stop tooth breakage^5^. Instead, we propose that the development of the deep folding increases the depth of the denticles within the tooth, likely strengthening each denticle, as does plicidentine in the tooth roots of some fish and amphibians^17^, creating a tooth microanatomy unique to theropods. In addition to the interdental folds, the presence of enamel spindles at the DEJ in each denticle likely lengthened the longevity of the serrated carinae^18^,^19^, given the high levels of tooth wear^28^ and slow tooth replacement rates with increased body size^29^ in theropod dinosaurs. True denticles typically last longer than simple enamel crenulations, which can wear away quickly after tooth eruption^30^. Replacement rates for large theropods are estimated to be over two years^29^, whereas replacement rates for varanids are only a few months^31^.

**Figure 7:**
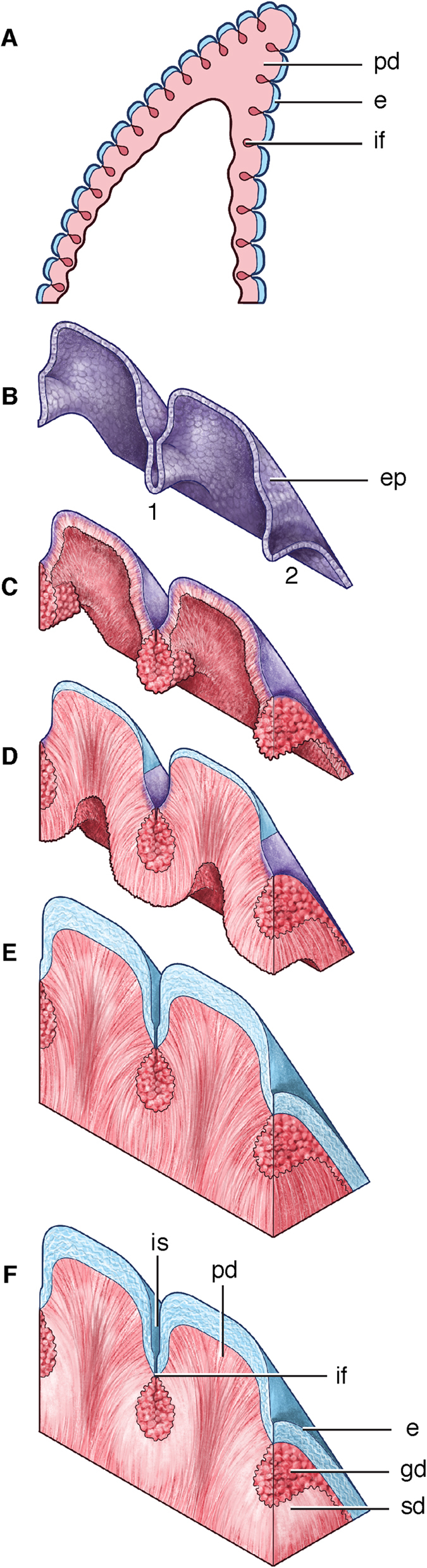
Proposed model for the development of theropod ziphodont teeth, drawn by Danielle Dufault. (**A**) Mineralization of the tooth tissues from tip towards the root. (**B**) Detail of two denticles, showing deposition of tooth tissues in 1) sagittal and 2) transverse views. Dental epithelium folds to form the shape of the tooth prior to differentiation of tooth tissues. (**C**) Dentine deposition by odontoblasts. Globular mantle dentine deposition in the interdental fold and the remainder of the tooth, and mantle dentine in the denticle tip. (**D**) Enamel deposition by ameloblasts at each denticle tip; primary dentine deposition. (**E**) Enamel mineralization into each interdental sulcus, stopping before closing the channel at the interdental fold in the dentine. Tooth eruption occurs at this stage. (**F**) Functional tooth with sclerotic dentine deposition. Abbreviations: e, enamel; ep, epithelium; if; interdental fold; is, interdental sulcus; pd, primary dentine; sd, sclerotic dentine. Colors: blue, enamel; purple, epithelium; red, dentine; white, sclerotic dentine.

Recent advances into the development of complex tooth shapes in reptiles^14^,^15^, and the deep homology of tooth tissues^32^, allows us to propose the first model for the development of ziphodont teeth (Fig. 7). In extant reptiles with multiple tooth cusps, amelogenesis begins at the tip of each cusp, and travels down the epithelium until joining with adjacent enamel^15^,^16^ (Fig. 7A). After the shape of the tooth is created by the epithelium (Fig. 7B), odontoblasts begin to deposit dentine pulp-ward, and ameloblasts deposit enamel in the opposite direction^16^. Differentiation of ameloblasts and enamel mineralization begins after the deposition of dentine begins (Fig. 7D). As in reptile tooth development, enamel deposition begins at the tips of the denticles and stops before abutting the enamel of the neighbouring denticle, creating the channel at each interdental sulcus, connecting the interdental fold to the exterior surface of the enamel (Fig. 7E). This pattern of enamel deposition may be controlled by an enamel bulge, as in living reptiles^14^. As the tooth becomes functional and ages, sclerotic dentine is deposited around the base of the interdental fold, likely in response to use of the tooth or aging (Fig. 7F).

This unique tooth microanatomy is present in taxa representing most major non-avian theropod groups, including the basal Triassic theropod *Coelophysis*, and we infer it as a synapomorphy of the clade. Ziphodont theropod dinosaurs thus evolved an apomorphic tooth development process that allowed for high serration densities^3^ and preserved the longevity of the tooth and its functionally important serrations. Interestingly, these characteristic structures of theropod teeth have been secondarily modified in two sampled taxa with proposed apomorphic feeding ecologies. *Troodon,* suggested to be omnivorous, herbivorous^33^, or at least preferred soft food items^34^ retains ziphodonty, but without the narrow spacing and deep interdental folds (Fig. 2F). True denticles are lost (Fig. 4C) in piscivorous spinosaurs^35^, which likely had small prey items relative to their body size. This suggests that the development of denticles was important for facilitating the particular hypercarnivorous feeding style of theropods, specifically, the ability to feed on large prey^36^,^37^ and to crush bone^27^,^38^.

## Materials and Methods

To examine the pervasiveness of ampullae in theropods, teeth from multiple taxa were examined. *Tyrannosaurus rex* (ROM 66108)*, Coelophysis bauri* (CM 76863 and 87671)*, Carcharodontosaurus saharicus* (ROM 52037)*, Troodon formosus* (ROM 05089)*, Allosaurus fragilis* (ROM 66106), and a tyrannosaurid (ROM 57981) were examined histologically. Unerupted teeth were taken from *Allosaurus* (UMNH.VP 23761), *Gorgosaurus* sp. (CMN 2225 and *Daspletosaurus* sp. RTMP 2012.012.0029). The teeth were prepared out of intact dentaries and maxillae, and had not erupted past the level of gumline. The *Allosaurus* tooth was completely unerupted, having not yet erupted past the level of the gumline, and the *Gorgosaurus*, *Daspletosaurus,* and other tyrannosaurid teeth were partially erupted and fully erupted. Other ziphodont animals examined include the shark *Carcharodon megalodon* (ROM 30530), from the Pleistocene of Florida, *Varanus komodoensis* (ROM R7565), an extant varanid lizard, *Dimetrodon grandis* (ROM 6039) from the Permian-aged Craddock Bonebed locality in Texas, USA, and a phytosaur (ROM 7981) from the Upper Triassic Howard County, Texas. Comparative material of *Smilodon* sp. (ROM 3288) from the Pleistocene of North America and a crocodilian (ROM 67512) from the Cretaceous Kem Kem beds of Morocco, *Triceratops* sp. (ROM 67669) from the Upper Cretaceous Hell Creek Formation, a hadrosaurid (ROM 58205) from the Cretaceous Oldman Formation, Alberta, and *Spinosaurus aegypticus* (ROM 65613) from the Cretaceous Kem Kem beds of Morocco were also cut.

Thin sections were prepared following standard histological methodology, with some modification^39^. Specimens were embedded in Castolite AP polyester resin, placed under vacuum and left to dry for 24 hours before being cut with a Buehler Isomet 1000 wafer blade low-speed saw. Specimens were mounted to frosted glass and plexiglass slides using Scotch-Weld SF-100 cyanoacrylate. Specimens were ground down to approximately 180 μm thick using a Hillquist grinding cup, then ground by hand using progressively finer grits of silicon carbide powder. Some specimens were polished using one-micron grit aluminum oxide powder. Specimens were photographed using a Nikon DS-Fi1 camera mounted to a Nikon AZ-100 and a Nikon EPOL 200 microscope with an oblique illumination slider. Images were processed using Nikon NIS-Elements (Basic Research) imaging software. Scanning Electron Microscope images were taken in a Jeol Neoscope JCM-5000 SEM, which does not require sputter coating. Non-histological photographs were taken with a Canon Rebel T3i camera.

### FTIR Synchrotron Analyses

The Fourier Transform Infrared spectroscopy (FTIR) analysis of a cf. *Gorgosaurus* sp. tooth (ROM 30582) was performed using synchrotron-radiation-based infrared microspectroscopy (SR-IMS) at BL14A1 of the National Synchrotron Radiation Research Center (NSRRC), Taiwan^40^ by employing an FTIR spectrometer (Nicolet 6700; Thermo-Nicolet Instruments, Madison, WI, USA) equipped with an infrared confocal microscope (Continuum; Spectra Tech Inc., Oak Ridge, TN, USA). The high collimation of the infrared synchrotron beam allows for a 20 × 20 μm^2^ spot to be focused on samples; this is superior to the 40 × 40 μm^2^ spot achievable using a conventional global beam^40^. The sample of fossil tooth was mounted onto Ag/SnO_2_-coated IR reflective low-e slides (Kevley Technologies, Chesterfield, OH, USA). The 1.5-GeV third-generation synchrotron was operating in top-up mode at 360 mA. The FTIR bench was configured with collimated infrared synchrotron radiation, providing external infrared synchrotron radiation to the FTIR spectrometer. The modulated synchrotron infrared light generated by the Michelson interferometer of FTIR spectrometer was directed into the infrared confocal microscope for microspectroscopy. The FTIR spectra were collected in the mid-infrared range of 4000–650 cm^−1^ at a spectral resolution of 4 cm^−1^ with the co-addition of 128 scans and an aperture of 20 × 20 μm^2^ and step size 10 μm. The optical systems of the FTIR microscope were purged by using dry nitrogen and an automatic atmospheric suppression function in OMNIC™ (OMNIC, 2001; Thermo Fisher Scientific, Waltham, MA, USA), which were employed to minimize infrared absorption by CO_2_ and water vapor in the ambient air. A single-beam background spectrum was collected from an area free of sample on low-e slide. Stage control and data collection were done using the spectrometer software coupled with a gating system that eliminated the noise during 60-s electron injection periods in top-up mode operation. Optical images were also obtained using a camera linked to the infrared confocal microscope.

### Institutional abbreviations

**CM**, Carnegie Museum, Pittsburgh, USA; **CMN**, Canadian Museum of Nature, Ottawa, Canada; **ROM**, Royal Ontario Museum, Toronto, Canada; **RTMP**, Royal Tyrrell Museum of Palaeontology, Drumheller, Canada; **UMNH**, Natural History Museum of Utah, Salt Lake City, USA.

## Additional Information

**How to cite this article**: Brink, K. S. *et al.* Developmental and evolutionary novelty in the serrated teeth of theropod dinosaurs. *Sci. Rep.* **5**, 12338; doi: 10.1038/srep12338 (2015).
